# Vasopressin and Its Analogues: From Natural Hormones to Multitasking Peptides

**DOI:** 10.3390/ijms23063068

**Published:** 2022-03-12

**Authors:** Mladena Glavaš, Agata Gitlin-Domagalska, Dawid Dębowski, Natalia Ptaszyńska, Anna Łęgowska, Krzysztof Rolka

**Affiliations:** Department of Molecular Biochemistry, Faculty of Chemistry, University of Gdańsk, Wita Stwosza 63, 80-308 Gdansk, Poland; mladena.glavas@ug.edu.pl (M.G.); dawid.debowski@ug.edu.pl (D.D.); natalia.ptaszynska@ug.edu.pl (N.P.); anna.legowska@ug.edu.pl (A.Ł.); krzysztof.rolka@ug.edu.pl (K.R.)

**Keywords:** vasopressin, vasopressin analogues, vasoconstrictors, vasopressin receptors, desmopressin

## Abstract

Human neurohormone vasopressin (AVP) is synthesized in overlapping regions in the hypothalamus. It is mainly known for its vasoconstricting abilities, and it is responsible for the regulation of plasma osmolality by maintaining fluid homeostasis. Over years, many attempts have been made to modify this hormone and find AVP analogues with different pharmacological profiles that could overcome its limitations. Non-peptide AVP analogues with low molecular weight presented good affinity to AVP receptors. Natural peptide counterparts, found in animals, are successfully applied as therapeutics; for instance, lypressin used in treatment of diabetes insipidus. Synthetic peptide analogues compensate for the shortcomings of AVP. Desmopressin is more resistant to proteolysis and presents mainly antidiuretic effects, while terlipressin is a long-acting AVP analogue and a drug recommended in the treatment of varicose bleeding in patients with liver cirrhosis. Recently published results on diverse applications of AVP analogues in medicinal practice, including potential lypressin, terlipressin and ornipressin in the treatment of SARS-CoV-2, are discussed.

## 1. Introduction

Homeostasis of living organisms requires continuous and strict regulation. The management of all body functions involves various mechanisms and components that need to be efficiently coordinated. All the distinct organs located within the body have to cooperate and respond to the changes in the environment very quickly and efficiently. For this reason, living organisms have developed highly specialized compounds keeping the body functions under tight control. Among them are peptides and proteins that act as hormones [1,2,3] and neurotransmitters [3] that take part in the defense system [4] by controlling respiration [5], metabolism, digestion, reproduction, etc.

In healthy organisms, under physiological conditions, all components of this complex network cooperate undisturbed. Pathological states, when some elements of this system fail, may result in various diseases, e.g., resistance to insulin results in Diabetes mellitus type 2 [6,7], while excess and aggregation of amyloid *β* peptide has a role in Alzheimer’s disease progress [8,9]. Currently, when the knowledge about the role and structure of biologically active peptides is pretty advanced, they are considered as potentially attractive therapeutics. Peptide synthesis is well developed [10,11] and peptide drug market is rapidly expanding [12,13]. Peptides occur naturally and are usually considered to be safer than synthetic drugs. They are also more selective and specific and, what is particularly important, they are degraded into nontoxic metabolites (amino acids) [14,15]. Taking it all into account, the introduction of peptides as drugs should be easy to handle.

However, despite their involvement in various bioactivities in living organisms and quite extensive knowledge regarding synthesis and structure–activity relationships, the utilization of peptides as drugs is not so straightforward [13,16]. In addition to indisputable advantages, peptides are not free from significant drawbacks [5,17]. The first major obstacle is problematic oral delivery, as most peptide drugs are characterized by low stability in the gastrointestinal tract. The short half-life of peptides is explained by the presence of peptidases in the body causing their degradation. The hydrophilic nature of peptides results in poor intestinal permeability [18,19] and, consequently, insufficient oral bioavailability. Currently, most peptide drugs are applied parenterally, which is normally considered as inconvenient. Oral application seems to be the easiest and most favorable way of taking medications [18]. Unfortunately, these are not the only problems with applying peptides in treatment. Their administration far from the target brings more biological barriers that drastically decrease peptides’ oral bioavailability.

Even though the utilization of peptides as drugs is challenging, their valuable properties make them extremely attractive candidates to become therapeutics. For this reason, modifications of peptide-based drug candidates increasing their metabolic stability and bioavailability, and retaining their functions are of high demand. Several significant achievements have been made, such as the introduction of non-proteinogenic amino acids, cyclization, modifications of termini or peptide bonds (pseudopeptides) and the design of peptide mimetics [13,15,20].

**Figure 1:**
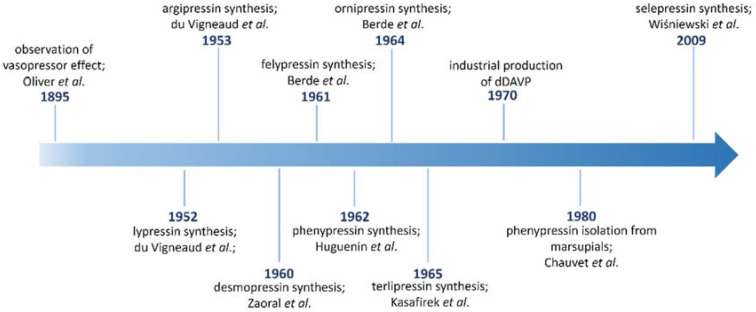
Timeline for development of AVP peptide analogues [28,29,30,31,32,33,34,35].

An obvious example of naturally occurring peptide with an especially valuable function in the body is vasopressin (AVP). This neurohormone is synthesized in overlapping regions in the hypothalamus in large magnocellular neurons situated in supraoptic nuclei and paraventricular nuclei [21,22]. In a healthy organism, AVP is responsible for the regulation of plasma osmolality by maintaining fluid homeostasis, e.g., it induces water reabsorption from urine and brings it back to circulation [23,24,25]. It is also a vasoconstricting agent taking part in blood pressure increase. In medical practice, it is used mostly in the treatment of vasodilatory shock; in septic shock during sepsis; and in the treatment of hypertension, edema and congestive heart failure [26]. After its discovery and subsequent chemical synthesis in 1953 [27], the next natural step was the urge to decipher its exact mechanism of action. For this reason, various AVP analogues, acting both as agonists or antagonists of its receptors, were designed and investigated. This review aims at presenting the most relevant ones. Figure 1 presents the milestones in the development of key peptide analogues of AVP, both of natural and synthetic origins.

## 2. Vasopressin

**Figure 2:**
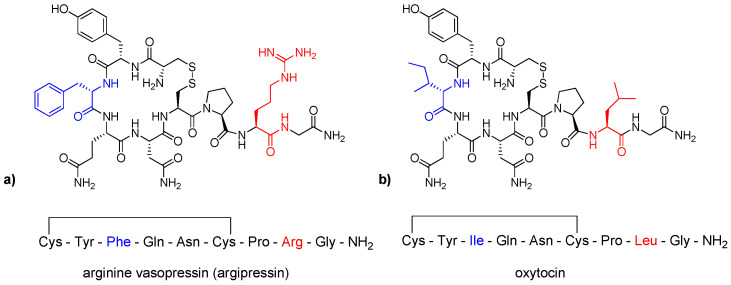
Structures of (**a**) arginine vasopressin (AVP) and (**b**) oxytocin (OXT). Differences in sequence are shown in blue and red.

Vasopressin (arginine vasopressin, argipressin, AVP, Figure 2a) is a nonapeptide comprising a tripeptide tail and a cyclic structure formed by six residues with a disulfide bridge between cysteines at positions 1 and 6 [36,37,38]. It is found exclusively in mammals and was isolated and synthesized for the first time in 1953 by Vincent du Vigneaud et al., who received the Nobel Prize in 1955 [27,39]. Its chemical structure is similar to another peptide hormone, oxytocin (OXT), which is the hydrophilic, cyclic nonapeptide (Cys(&)-Tyr-Ile-Gln-Asn-Cys(&)-Pro-Leu-Gly-NH_2_, Figure 2b) [38,40,41] composed of a six-membered ring with a disulfide bridge and tripeptide tail. The main differences are at positions 3 (Phe (AVP) → Ile (OXT)) and 8 (Arg (AVP) → Leu (OXT)) [42]. OXT was found in mammals in 1906. It is synthesized as inactive protein precursor of OXT [41] and active OXT is released from the posterior pituitary gland. OXT is also found in multiple organs, including heart, placenta, uterus and testes [40]. Its key role is the modulation of social bonding, emotions, maternal behavior, etc. [41,43]. AVP is synthesized from a precursor protein named preprovasopressin, which consists of AVP, neurophysin II and copeptin [44]. They are packed into neurosecretory vesicles and transported to the nerve endings located in neurohypophysis [27,45].

**Figure 3:**
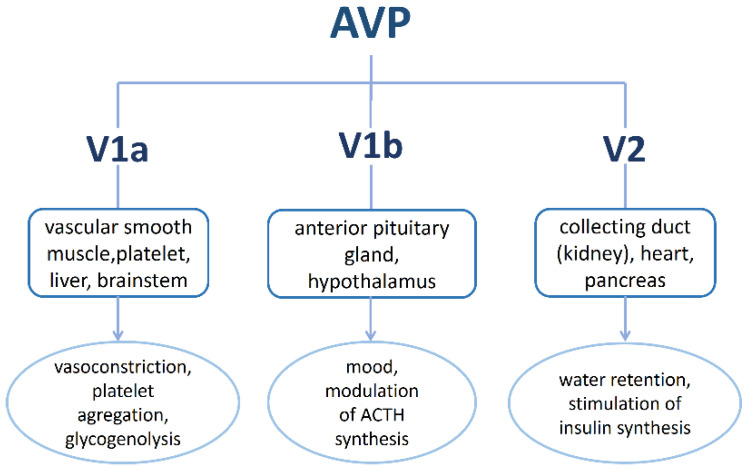
Schematic presentation of AVP receptors, their position and role in the body.

AVP binds to three G protein-coupled receptors: V1a (Avpr1a or V1), V1b (Avpr1b or V3) and V2 (Avpr2) [46]. They all share a high primary sequence homology, but have great diversity in their functional properties (Figure 3) [47]. V1a is a vascular receptor found primarily in vascular smooth muscle cells but was also discovered in platelets, liver, blood vessels, renal mesangial cells, brain, uterus and adrenal cortex [48,49,50,51]. Activated V1a receptor controls blood pressure, glycogenolysis and contraction of mesangial cells. It also facilitates vasoconstriction through the activation of phospholipase A, C and D, which results in the mobilization of intracellular calcium ions (Ca^2+^) [21,49] and leads to platelet aggregation (procoagulant property) [52]. Myocardial infarction and stress increase V1a receptor expression, while the activation of V1a in the brain can exacerbate brain edema [49]. V1b receptor is not only expressed in the anterior pituitary [21,52] but also in peripheral tissues such as the pancreas, thymus, lungs, heart and kidneys. It controls the excretion of an adrenocorticotropic hormone (ACTH) [21,23,53] and regulates cardiovascular and adrenal functions by mediating catecholamine excretion. It also participates in the modulation of behavior in social interactions and in the memory process [21]. Together with V1a, it activates the phosphatidylinositol hydrolysis pathway (formation of 1,2-diacylglycerol and inositol-1,3,4-triphosphate) and mobilizes intracellular Ca^2+^ [49,54]. AVP receptor V2 is found in the kidneys where it is expressed on basolateral surface of renal tubular cells [45,52,55,56]. The main role of activated V2 receptor is water reabsorption from the urine, i.e., reduction in urine volume (antidiuretic effect) [21,26,49]. The mechanism of water reabsorption is based on changes in plasma osmolality [57]. This results in electrical simulation (depolarization of cells) and the production of AVP [21,27]. AVP binds to V2 receptor expressed in the basolateral side of principal cells responsible for the regulation of water permeability in collecting duct of the kidney. This activates the adenylate cyclase and the production of cyclic adenosine monophosphate (cAMP). The latter further activates protein kinase (PKA) that phosphorylates some of the *C*-terminal residues of aquaporin-2 (AQP-2). In effect, AQP-2 is inserted into the apical side of principal cell-forming AQP-2 water channels. Water is reabsorbed via these channels into the principal cells and exits through AQP-3 and AQP-4 water channels to the interstitium. In this manner, water is reabsorbed from urine into the blood [22,45,52,58,59,60,61]. In addition to this classic PKA/cAMP-dependent pathway, cAMP functioning might also be mediated by the exchange of protein directly activated by cAMP [62]. The translocation of AQP-2 from the basolateral to apical regions of cells is dependent on AVP-induced Ca^2+^ mobilization. Thus, Ca^2+^ mobilization is crucial for water permeability in the inner medullary collecting duct. This process may be blocked with intracellular Ca^2+^ chelation with 1,2-bis(2-aminophenoxy)ethane-*N*,*N*,*N*,*N*-tetraacetic acid (BAPTA) [63]. The process of plasma osmolality increase/decrease is so sensitive that even a change of 1% can alter AVP release/inhibition [57]. After the release of AVP, only 10–20% of the total amount is present in blood circulation and its plasma half-life is 6–20 min [24,52,64]. The lack of AVP results in diabetes insipidus, which means that no other hormone can replace AVP [24]. The expression of V2 was also detected in central nervous system (CNS) where its presence was associated with anxiety, development, alcohol preference and aggression [26,49].

Release of AVP can be induced under all types of stress—immunological, physical and emotional [49,65]. The concentration and secretion of AVP may be regulated by insulin [45], changes in plasma osmolality (decrease in serum osmolality reduces AVP secretion, while an increase in serum osmolality promotes the secretion of AVP) [57,66] or use of morphine, alcohol and nicotine [57]. Depending on the various external factors influencing the body and its actual internal needs, AVP is able to regulate numerous biological processes. They include the following: increase in cortisol concentration through the induction of corticotropic axis stimulation [52], insulin secretion, release of coagulant factors [45], regulation of lipid metabolism [56], stimulation of water reabsorption, regulation of glucose mechanism, platelet aggregation, modulation of neuronal functions [54], growth of surface placental leucine aminopeptidase (P-LAP; enzyme crucial to maintain pregnancy) level [67], rise of plasma osmolality, regulation of blood pressure and volume [66] and reduction in arrhythmias and tachycardia [26]. AVP has also been shown to affect modulation of fear and memory [27], maintain cardiovascular homeostasis through vascular smooth muscle cell contraction [68], regulate pancreatic islet function [46], reduce hemorrhage [69] and, together with angiotensin II, preserve fluid and electrolyte balance [70]. Its expression in CNS has an impact on a variety of brain functions such as social interaction and recognition, aggression, maternal behavior, pair-bonding behavior and depression [21]. Although its role is irreplaceable, AVP frequently induces severe side effects because of its systemic vasoconstricting effect. This, in turn, induces an increase in peripheral resistance, with a reduction in cardiac output and heart rate and ultimately of coronary blood flow. These hemodynamic effects may result in myocardial ischemia or infarction, cardiac arrhythmias, mesenteric ischemia, ischemia of the limbs and cerebrovascular accidents [71]. As far as the trade market is concerned, AVP is marketed under different names in different countries worldwide. In the Unites States of America, it is used under the international name Pitressin or Pressyn [27], while in Spain it can be found under the name Vasostrict [72]; in India as Cpressin, Cevas-40, Cpressin P, etc. [73]; in Germany as Empressin [74]; and in France under the name Reverpleg [75].

### 2.1. Vasopressin Analogues

Despite its significant role in the treatment of various diseases, such as diabetes insipidus [47], vasodilatory shock and hypertension [26], the use of AVP is associated with some serious limitations, including short biological half-life, lack of specificity for V1 and V2 receptor and side effects [71,76]. AVP can often induce hyponatremia, decrease cardiac output and platelet count or increase the direct and indirect bilirubin level in the blood. The efforts to solve some of these shortcomings and discover new analogues characterized by better activity and selectivity [38] constitute a very important area of research. In 1980, AVP receptors (V1a, V1b and V2) were cloned and characterized [21], which was a milestone in the development of new AVP analogues. In the last forty years, various peptide and non-peptide analogues, acting as agonists or antagonists of these receptors, were designed and synthesized [47]. Nevertheless, analogues that could be very active, potent and truly selective for AVP receptors still remain an area of great interest. Such analogues constitute a pharmacological toolbox essential to effectively study the mechanism of action of AVP and ligand–receptor interactions [77].

#### 2.1.1. Non-Peptide Synthetic Analogues of AVP

**Figure 4:**
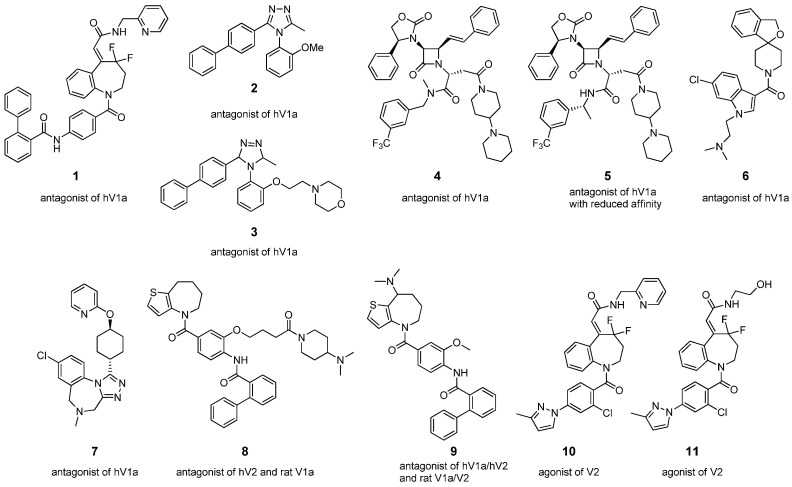
Non-peptide agonists and antagonists of AVP receptor.

The synthesis of small molecules mimicking larger compounds is of great interest and a great challenge in chemistry, especially when it comes to non-peptide molecules intended to imitate peptides. In the last few years, some low molecular weight AVP analogues were synthesized and presented good affinity to AVP receptors. Such non-peptide agonists and antagonists of AVP receptors are presented in Figure 4. Compound **1** had a high affinity to human V1a receptor (hV1a) and potently inhibited AVP-induced physiological responses of human coronary artery smooth muscle cells (CASMC) [68]. Compounds **2** and **3** showed high binding efficiencies to the hV1a receptor [78] and a similar chemical structure based on 4,5-diphenyl-1,2,4,-triazole moiety. Interestingly, compound **4** revealed high in vitro hV1a antagonistic affinity, while compound **5** had reduced hV1 binding affinity. This suggests that *α*-methyl substitution shifted the binding mode of the adjacent phenyl ring [79]. Compound **6** was a potent and selective brain-penetrant hV1a antagonist as well as **7** (belovaptan), which demonstrated an amelioration of socialization and communication [80]. Belovaptan underwent four clinical tests for use in the treatment of autism spectrum disorder, but only one was completed, while two were terminated and one was withdrawn [81]. Analogue **8** resulted in good bioavailability and extremely strong binding to the rat V1a and hV2 receptor. It had a potent diuretic effect and showed antagonism of AVP-induced hypertension [82]. Hydrochloride **9** showed high selectivity for rat V1a/V2 and hV1a/hV2 receptors and was under the clinical testing for patients with edema [83,84]. Compounds **10** and **11** were V2 receptor agonists characterized by high binding activity [50,61].

#### 2.1.2. Natural Peptide Analogues of Vasopressin

##### Lypressin

**Figure 5:**
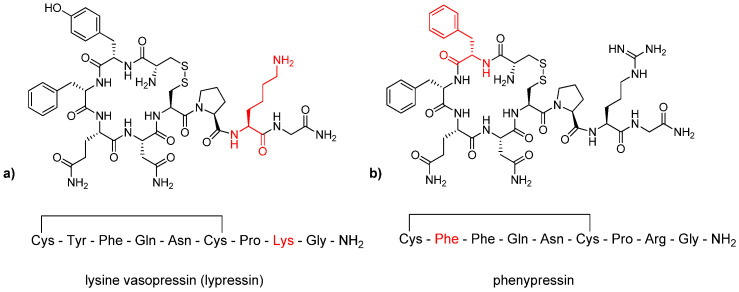
Natural peptide analogues of AVP, (**a**) LVP and (**b**) phenypressin. Amino acids that are not present in AVP are shown in red.

Lypressin (lysine vasopressin, LVP; Figure 5a) differs from AVP by one amino acid, i.e., Lys instead of Arg at position 8 (Table 1) [64]. The disulfide stretching bands in the Raman spectra of LVP exhibited small shoulders, which indicates the presence of some conformational flexibility in the disulfide moiety. Likewise, the circular dichroism spectra reveal the presence of more than one conformation of the disulfide unit [85].

LVP is the porcine antidiuretic hormone [86], and its plasma half-life is 5–7 min [64]. Disulfide bridge may be reduced, which leads to a loss of biological activity [87]. LVP is used to treat diabetes insipidus and to improve vasomotor tone and blood pressure [86]. It is available for clinical use as a nasal spray [88]. Such an application form exerts its efficiency up to eight hours, and patients treated with it are free of allergic reactions. LVP does not cause any elevation of blood pressure and has been proved to be safe for use during pregnancy and parturition [89].

**Table 1:** Comparison of AVP with its natural peptide analogues. Amino acid residues that are not present in AVP are marked in bold.

Analogue	Sequence	Source	Main Application	Refs.
Arginine vasopressin, argipressin, AVP	Cys(&)-Tyr-Phe-Gln-Asn-Cys(&)-Pro-Arg-Gly-NH_2_	human and other mammals	antidiuretic effect, maintenances cardiovascular homeostasis, increases blood pressure in septic shock	[38,44,45,46,49,65]
Lysine vasopressin, lypressin, LVP	Cys(&)-Tyr-Phe-Gln-Asn-Cys(&)-Pro-**Lys**-Gly-NH_2_	pigs	antidiuretic agent, hemostatic, vasoconstrictor agent	[64,86,87]
Phenypressin	Cys(&)-**Phe**-Phe-Gln-Asn-Cys(&)-Pro-Arg-Gly-NH_2_	marsupials (gray and red kangaroo, tammar and quokka wallaby)	increases the reabsorption of water in the kidneys and blood pressure	[90,91]

**Figure 6:**
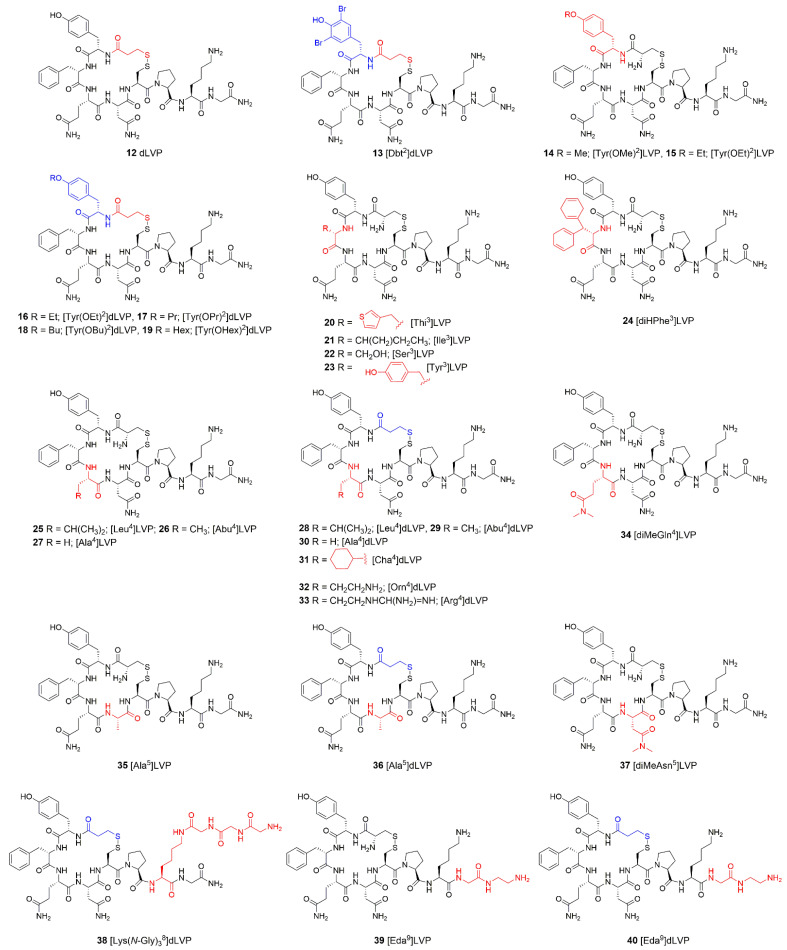
Structure of peptide LVP analogues. The modifications are shown in blue and red. d = deamination of *N*-terminal Cys (Cys1); Dbt = 3,5-dibromo-l-tyrosine; Thi = thienilalanine; diHPhe = dihydrophenylalanine; Abu = 4-*α*-aminobutyric acid (AABA); Cha = 1-amino-cyclopentanecarboxylic acid (cyclohexylalanine); Eda = ethylendiamine.

Since 1960, many LVP analogues have been synthesized. The effect of modifications in the side chains of various amino acids on the biological activity has been examined. The most relevant analogues and the results of their biological investigation, including antidiuretic and vasopressor activities, are presented in Figure 6 and in Table 2.

Jarial et al. [92] compared the diagnostic accuracy of LVP and human corticotrophin releasing hormone) as stimulating agents for ACTH release during bilateral inferior petrosal sinus sampling (BIPSS) to localize and lateralize the source of ACTH in patients with Cushing’s syndrome (CS). The results showed that LVP stimulating ACTH secretion in BIPSS test for CS and 10 units LVP correctly lateralized the pituitary adenoma in three-fourths of patients. BIPSS was suggested to be used as a good method to identify ACTH-secretion sources [93].

**Table 2:** Peptide LVP analogues and their biological activity.

Peptide	Antidiuretic Activity Units/mg	Vasopressor Activity Units/mg	Oxytocic Activity Units/mg	Other Activities and Comments
LVP	203 ± 7 [94] 240 ± 13 [95,96]	243 ± 3 [94] 266 ± 18 [96]	7.3 ± 0.2 [96] 4.8 ± 0.3 [94]	AVD ^a^ = 48 ± 2 units/mg [94]
dLVP (**12**) ^b^	301 ± 11 [96] 550 ± 1.7 [97]	126 ± 2 [96] 145 ± 7 [97]	12 ± 0.5 [96,97]	-
[Dbt^2^]dLVP (**13**) ^c^	nr ^d^	nr	nr	AVD = *p*A_2_ = 6.97 ^e^; M = 1.1 × 10^−7^; no measurable antagonistic, oxytocic and pressor activities [98]
[Tyr(OMe)^2^] LVP (**14**)	1.5–3 [99]	79 [99]		antioxytocic and antipressor properties, antagonistic character of these analogues results from the bulky, lipophilic substituents on the aromatic ring rather than from the blocking or elimination of the phenolic group [99]
[Tyr(OEt)^2^] LVP (**15**)	nr	5 [99]		
[Tyr(OX)^2^] dLVP (**16**–**19**) ^f^	0.5–2.0 units/μmol [100]	0.5–3.0 units/μmol [100]		weak agonistic properties; in the rat, none of the analogues inhibited the antidiuretic action of LVP when the two substances were administered together in a single injection; completed inhibition was obtained when X = Et; antagonistic potency decrease with increasing size of alkyl substitution [100]
[Thi^3^]LVP (**20**)	332 ± 32 [101,102]	243 ± 5 [101,102]	19 ± 0.5 [101,102]	AVD = 87 ± 4 units/mg; steric size in position 3 plays significant role in the manifestation of vasopressin-like activities [101,102]
[Ile^3^]LVP (**21**)	24 ± 3 [101,102]	130 ± 13 [101,102]	78 ± 10 [101,102]	AVD = 210 ± 3 units/mg [101,102]
[Ser^3^]LVP (**22**)	~0.08 [102]	<0.01 [102]	nr	uterotonic activity ≤ 0.01 units/mg [102]
[Tyr^3^]LVP (**23**)	0.18 [102]	1.6 [102]		
[diHPhe^3^] LVP (**24**)	125–130 [102]	129–132 [102]		uterotonic activity = 6 units/mg; effective agonist; position 3 is not very restrictive for vasopressor receptors and antidiuretic potency [102]
[Leu^4^]LVP (**25**)	1–2 [95]	1.33 [95]	negligible [95]	AVD = 1 unit/mg [95]
[Leu^4^]dLVP (**28**)	5–6 [95] 10.5 ± 0.9 [97]	0.55 [95] 0.9 ± 0.1 [97]	0.054 ± 0.002 [97]	AVD = 4.60 units/mg [95] high affinity for the rat V1b receptor, very low affinities for the rat V1a and V2 receptor, potent agonists for the rat V1b receptor, weak agonists for the rat antidiuretic activity [97], Gln is not essential for biological activity [95]
[Abu^4^]LVP (**26**)	707 [95]	nr	nr	Gln is not essential for biological activity [95]
[Abu^4^]dLVP (**29**)	729 [95]	3.5 [95]		
[Ala^4^]LVP (**27**)	707 ± 107 [96]	10.2 ± 0.6 [96]	1.54 ± 0.1 [96]	-
[Ala^4^]dLVP (**30**)	729 ± 26 [96]	3.5 ± 0.2 [96]	1.51 ± 0.05 [96]	
[Cha^4^]dLVP (**31**)	0.82 ± 0.01 [97]	0.043 ± 0.008 [97]	*p*A_2_ = 6.48 ± 0.03 M = 3.41 × 10^−7^ ± 0.2 [97]	high affinity for the rat V1b receptor, very low affinities for the rat V1a and V2 receptor, potent agonists for the rat V1b receptor, weak agonists for the rat antidiuretic activity [97]
[Orn^4^]dLVP (**32**)	7.8 ± 0.4 [97]	0.23 ± 0.02 [97]	3.1 ± 0.1 [97]	-
[Arg^4^]dLVP (**33**)	784 ± 54 [97]	83 ± 4 [97]	0.15 ± 0.02 [97]	
[diMeGln^4^] LVP (**34**)	1.88 ± 0.04 [94]	1.27 ± 0.03 [94]	<0.05 [94]	AVD ≤ 0.1 units/mg [94]
[Ala^5^]LVP (**35**)	~0.2 [96]	0.15 ± 0.01 [96]	<0.001 [96]	carboxamide group is essential for activity [96]
[Ala^5^]dLVP (**36**)	~0.05 [96]	~0.015 [96]	<0.002 [96]	
[diMeAsn^5^] LVP (**37**)	5.5 ± 0.3 [103]	2.55 ± 0.05 [103]	<0.05 [103]	AVD = 0.39 ± 0.03 units/mg; hydrogen atoms of carboxamide group are not essential for antidiuretic activity [103]
[Lys(N-Gly)_3_^8^]dLVP (**38**)	nr	nr	nr	more powerful and prolonged analgesia compared to LVP [104]
[Eda^9^]LVP (**39**)		<0.05; 0.002 [105]		-
[Eda^9^]dLVP (**40**)		126 [105]		

^a^ AVD = avian vasodepressor; ^b^ d = deamino cysteine (Cys1); ^c^ Dbt = 3,5-dibromo-l-tyrosine; ^d^ nr = not reported; ^e^ *p*A_2_ values in vitro represent the negative logarithm to the base 10 of the average molar concentration (M) of the antagonist that reduces the response to 2x units of agonist to equal the response seen with x units of agonist administered in the absence of the antagonist; ^f^ X = ethyl- (Et), propyl- (Pr), butyl- (Bu), hexyl- (Hex).

##### Phenypressin

Phenypressin ([Phe^2^]AVP, Figure 5b) is another neuropeptide belonging to the vertebrae vasopressin family [90]. It has been found mostly in some species of the Macropodidae family, particularly in eastern gray kangaroo, red kangaroos, tammar wallaby and the quokka wallaby [31,34,91,106]. Phenypressin, similarly to AVP, is synthesized in the hypothalamus and travels to the posterior pituitary and is then released into the vesicles. Phenypressin was found to be less abundant than vasopressin-like peptides in marsupials [106]. The name phenypressin was suggested by Chauvet et al. [34] because Tyr2 in AVP is replaced by Phe (Table 1) and was synthesized for the first time in 1962 by Huguenin and Boissonnas [31]. The comparison of the pharmacological properties of phenypressin with those of AVP revealed a reduction in rat antidiuretic activity from 435 to 375 units/µmol and rat blood pressure activity from 435 to 130 units/µmol [107].

#### 2.1.3. Synthetic Peptide Analogues of Vasopressin

Numerous peptide analogues of AVP have already been synthesized. They are based mostly on the changing of one or more amino acids in its sequence or side chain modification such as *N*-methylation, *α*-alkylation and local or global cyclization. Extensive research on AVP analogues allowed the determination of the role of specific residues in AVP biological activity. Table 3 presents selected analogues of this hormone described in this section together with their vasopressor, antidiuretic and oxytocic potencies.

**Figure 7:**
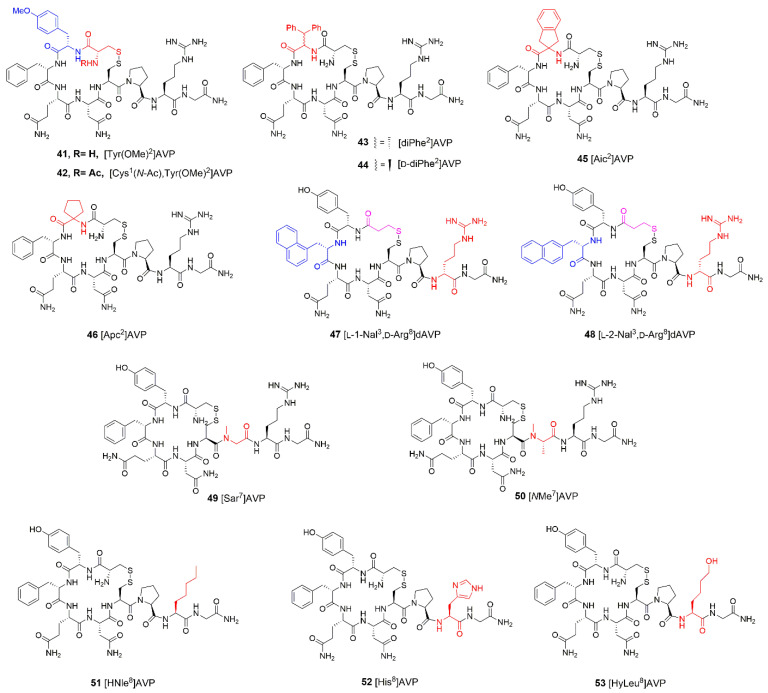
Structures of selected synthetic peptide analogues of AVP. Modifications are shown in different colors. d = deamination of *N*-terminal cysteine (Cys1); diPhe = 3,3-diphenyl-l-alanine; d-diPhe = 3,3-diphenyl-d-alanine; l-1-Nal = l-1-napthylalanine; l-2-Nal = l-2-napthylalanine; Aic = 2-aminoindane-2-carboxylic acid; Apc = 1-aminocyclopentane-1-carboxylic acid; Sar= l-sarcosine; NMeAla= N-methyl-l-alanine; HNle = l-homonorleucine; HyLeu = l-hydroxynorleucine.

Tyr at position 2 is considered to contribute to the initiation of the pressor response to AVP [51,108]. Indeed, modification in Tyr2 of AVP with methyl group (Me) ([Tyr(OMe)^2^]AVP], **41**, Figure 7) resulted in an analogue with decreased vasopressor potency and higher antidiuretic activity [109]. The acetylation of its free amine group of Cys ([Cys^1^(*N*-Ac),Tyr(OMe)^2^]AVP, **42**, Figure 7) reduced antidiuretic activity 15,000 times [110]. Tyr 2 replacement with 3,3-diphenyl-l-alanine ([diPhe^2^]AVP, **43**, Figure 7) or its d-enantiomer (**44**, Figure 7) increases antidiuretic activity, which is even more remarkable in the case of [d-diPhe^2^]AVP. Vasopressor potency of both analogues was completely abolished [111,112]. Modification at position 2 with 2-aminoindane-2-carboxylic acid (Aic, [Aic^2^]AVP, **45**, Figure 7) did not alter the interaction with the receptor and preserved antidiuretic activity similar to AVP [77], while substitution of position 2 with 1-aminocyclopentane-1-carboxylic acid ([Apc^2^]AVP, **46**, Figure 7) resulted in higher antidiuretic activity [56].

Phe at position 3 is important for binding to the receptor and determines hormone antidiuretic and uterotonic activity [51]. Its replacement with l-1-naphthylalanine (l-1-Nal), inversion of configuration at position 8 and deamination of the *N*-terminus yield the [l-1-Nal^3^, d-Arg^8^]dAVP, **47**, Figure 7) potent agonist of V2 receptor. Interestingly, when l-1-Nal in position 3 is replaced by l-2-Nal (**48**, Figure 7), the selective V1 receptor antagonist is obtained [113].

Pro7 has a key role in the orientation of tripeptide tails and in the conformational constraints of peptide and binding to V2 receptor [114,115]. Analogues with Pro7 replaced by l-sarcosine ([Sar^7^]AVP, **49**, Figure 7) or *N*-methyl-l-alanine ([*N*MeAla^7^]AVP, **50**, Figure 7) turned out to be specific antidiuretic agents, while their vasopressor activity was abolished. They also retained high binding affinities relative to renal vasopressin receptors V2, but the binding affinity to V1 receptor was lost [114]. Deletion of Pro retained antidiuretic antagonist activity. Pro7 was reported to be important for binding and activation of the V2 receptor, whereas it was not necessary for V2 receptor antagonistic activity [115].

To examine the importance of the basic amino acid at position 8 relative to AVP and LVP biological activity, the analogue with l-homonorleucine (HNle) was synthesized [116]. The results showed that [HNle^8^]AVP (**51**, Figure 7) has 8% of LVP activity. Its antidiuretic activity in ethanol-anesthetized rats equals about 10 units/mg, as compared to about 250 units/mg for LVP. These results indicate that amino group in position 8 is not indispensable in terms of showing vasopressin-like activities, although a cation-forming group is needed for potencies comparable to those of the natural hormone. Interestingly, any basic residue in this position is not sufficient for high potency. This was confirmed by the example of [His^8^]AVP (**52**, Figure 7), which showed about 270-fold and 130-fold decrease in pressor activities when compared to AVP and LVP, respectively [117]. The relative basicity of amino acid residue has a considerable influence on the degree of pressor activity. To examine if the hormone is bound to the receptor and not through an ionic bond but by a hydrogen bond, AVP, an analogue with l-hydroxynorleucine [HyLeu^8^]AVP, **53**, Figure 7) in position 8 was synthesized. Its hormonal activities are moderate but were significantly higher than those of [HNle^8^]AVP. This suggests that the hydrogen bond was able to substitute, to some extent, the ionic bond between hormone and receptor. Alternative explanations, such as polar interactions or hydrophilic effect, cannot be ignored [116].

**Table 3:** Synthetic peptide AVP analogues and their biological activity.

Peptide	Antidiuretic Activity Units/mg	Vasopressor Activity Units/mg (for Agonist) *p*A_2_ (for Antagonist)	Oxytocic Activity Units/mg (for Agonist) *p*A_2_ (for Antagonist)
AVP (Figure 2a)	323 [118] 450 (*t*_1/2_ = 60) 450 (*t*_1/2_ = 200) [119]	369 [118] 412 [119]	14 [118]
[Tyr(OMe)^2^]AVP (**41**)	386 ± 36 [109]	*p*A_2_= 9.7 ± 0.5 ^a^ [109]	*p*A_2_ = 7.44 ± 0.12 (no Mg^2+^) and 6.34 ± 0.19 (in 0.5 mM Mg^2+^) [109]
[Cys^1^(*N*-Ac), Tyr(OMe)^2^]AVP (**42**)	0.026 ± 0.002 [110]	*p*A_2_= 7.18 ± 0.08 [110]	*p*A_2_ = 7.29 ± 0.08 (no Mg^2+^) and 6.73 ± 0.14 (in 0.5 mM Mg^2+^) [110]
[diPhe^2^]AVP (**43**)	450 (*t*_1/2_ = 60) 9000 (*t*_1/2_ = 200) [112]	0 [112]	*p*A_2_ = 7.00 ± 0.20 [112]
[d-diPhe^2^]AVP (**44**)	1000 (t_1/2_ = 60) 45,000 (*t*_1/2_ = 200) [111,112]	0 [111]	*p*A_2_ = 7.82 ± 0.39 [111]
[Aic^2^]AVP (**45**)	450 (*t*_1/2_ = 60) 45,000 (*t*_1/2_ = 200) [77]	9.4 ± 2.8 [77]	*p*A_2_ = 7.27 ± 0.22 (no Mg^2+^) [77]
[Apc^2^]AVP (**46**)	1800 (*t*_1/2_ = 60) 1800 (*t*_1/2_ = 200) [56]	13.4 ± 3.8 [56]	0.2 units/mg *p*A_2_ = 6.0 (no Mg^2+^) [56]
[l-1-Nal^3^, d-Arg^8^]dAVP (**47**)	2.2 ± 0.83 [113]	nr ^b^	nr
[l-2-Nal^3^, d-Arg^8^]dAVP (**48**)	3.79 ± 1.31 [113]	nr	weak [113]
[Sar^7^]AVP (**49**)	188 ± 19 [114]	3.6 ± 0.2 [114]	nr
[NMeAla^7^]AVP (**50**)	343 ± 54 [114]	10.6 ± 0.4 [114]	nr
[HNle^8^]AVP (**51**)	10 [116]	21.4 ± 1.0 [116]	nr
[His^8^]AVP (**52**)	nr	1.5 [117]	nr
[HyLeu^8^]AVP (**53**)	70 [116]	30 [116]	nr

^a^ *p*A_2_ values in vitro represent the negative logarithm to the base 10 of the average molar concentration (M) of the antagonist that reduces the response to 2x units of agonist to equal the response seen with x units of agonist administered in the absence of the antagonist; ^b^ nr = not reported; *p*A_2_ values represent the negative logarithm to the base 10 of the average molar concentration of an antagonist that reduces the response to 2x units of the agonist to x units of the agonist.

##### Desmopressin

**Figure 8:**
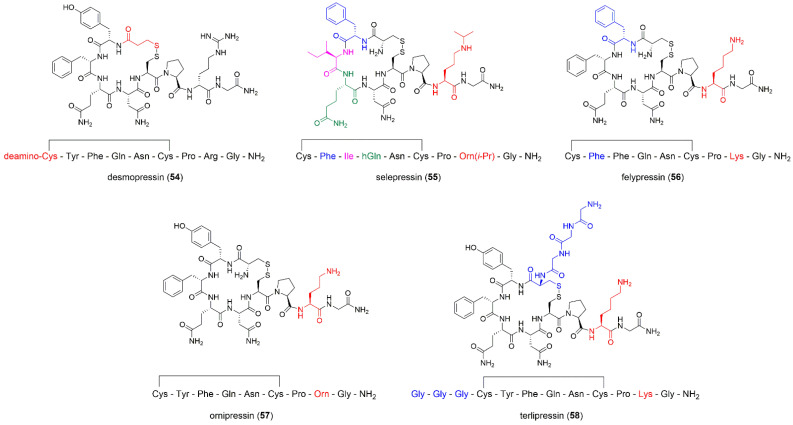
Synthetic peptide analogues of AVP. Modifications are shown in different colors. hGln = homoglutamine, Orn(*i*-Pr) = *N*-*δ*-*iso*-propyl-l-ornithine.

Desmopressin (1-desamino-8-d-Arginine vasopressin, 1-deamino-8-d-arginine vasopressin, dDAVP; **54**, Figure 8) is a synthetic, deaminated analogue of AVP, bearing the substitution in position 8, where d-Arg replaces l-Arg (Table 4) [120,121]. As compared to AVP, dDAVP has a longer plasma half-life of 90–190 min [64] and is more resistant to degradation by pancreatic proteases (mostly by trypsin) and has superior affinity to V2. dDAVP is referred to as the first generation analogue of vasopressin. The first chemical synthesis of dDAVP was conducted in a solution in 1967 [122,123]. The introduction of solid phase peptide synthesis (SPPS) strategy enabled its production on a bigger, commercial scale [124].

dDAVP was subjected to several experiments assessing structure–activity relationships (SARs). Nearly all amino acid residues were successively substituted with Ala, except both Cys residues located at positions 1 and 6, which are responsible for cyclization. It was demonstrated that *N*-terminal amino acids located at the peptide’s loop play a crucial role in antiproliferative activity against the aggressive MDAMB231 human breast cancer cell line [139]. Substitutions in the positions 2, 3 and 5 caused the highest reduction (50–60%) in cytostatic effects. Such an observation is in accordance with a molecular modelling study [140] and other previous research demonstrating the pivotal role of vasopressin’s *N*-terminal end in binding with V2 receptor [141,142]. The substitution of Gln4 and Gly9 was more tolerable and caused a 30% reduction in activity. On the other hand, the replacement at *C*-terminal position 7 (Pro replaced either with Ala or (2*S*,4*R*)-*p*-hydroxyproline (Hyp)) and 8 (d-Arg) did not affect the antitumor effect.

**Figure 9:**
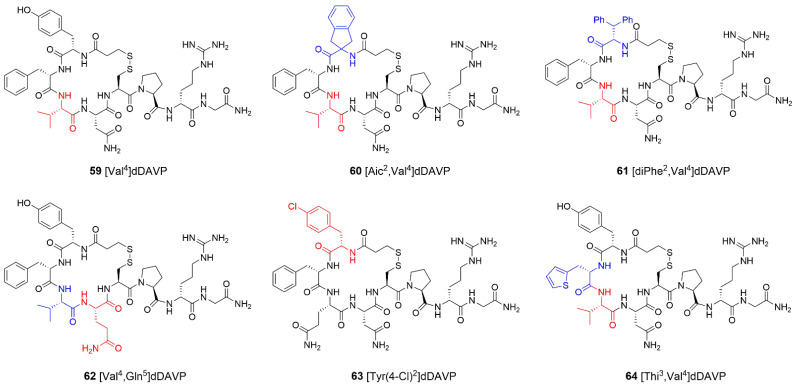
Analogues of dDAVP. Modifications are shown in different colors. Aic = 2-aminoindane-2-carboxylic acid; diPhe = 3,3-diphenyl-l-alanine; Thi = thienilalanine.

As shown by Sawyer et al. [143], a single substitution at position 4 (replacement of Gln with Val, **59**, Figure 9) resulted in dDAVP analogue with undetectable vasopressor action and antidiuretic activity about four times that of AVP. As compared to dDAVP, [Val^4^]dDAVP had about 10-fold higher affinity, expressed as the *K*i value, to human V2 receptor. However, similarly to dDAVP, it was also a potent agonist of the human V1b receptor [141]. It was demonstrated recently that [Val^4^]dDAVP could rescue the function of the N321K-mutated V2 receptor without significant side effects on the V1-initiated vasoconstriction [144]. [Val^4^]dDAVP had a similar potency as AVP to stimulate the production of cAMP by this mutant receptor but, in contrast to the natural hormone, it did not promote vasoconstriction of peripheral mouse arterioles. Thus, the administration of [Val^4^]dDAVP may be considered as beneficial for patients with nephrogenic diabetes insipidus. The substitution of Tyr2 with bulky, sterically restricted Aic ([Aic^2^,Val^4^]dDAVP, **60**, Figure 9) resulted in peptides with high antidiuretic activity comparable to that of dDAVP [66]. Similarly, the introduction of bulky diPhe ([diPhe^2^,Val^4^]dDAVP], **61**, Figure 9) or d-diPhe in position 2 of [Val^4^]dDAVP generated exceptionally potent antidiuretic agents in rats with significantly prolonged activities [112].

Another [Val^4^]dDAVP analogue, in which Asn5 was replaced with Gln (**62**, Figure 9), has been studied extensively as a potential anticancer agent [145]. It was demonstrated that [Val^4^,Gln^5^]dDAVP had significant antiproliferative activity against V2-receptor expressing MCF-7 human breast carcinoma cell line [145,146]. As compared to dDAVP, [Val^4^,Gln^5^]dDAVP exerted superior inhibitory effect on breast cancer cell proliferation and colony formation, reduced tumor growth and angiogenesis and improved the survival rate in the triple-negative MDA-MB-231 xenograft in nude mice [147]. Treatment with [Val^4^,Gln^5^]dDAVP caused a complete inhibition of metastatic progression in hormone independent and metastatic F3II breast cancer mouse model [148]. The combination of [Val^4^,Gln^5^]dDAVP either with chemotherapeutic agent paclitaxel in treatment of MDA-MB-231 tumor bearing nude mice or with carmustine in metastatic F3II breast cancer model resulted in greater tumor growth inhibition as compared to single-drug regimen [147]. Moreover, the beneficial effect involved a reduction in local aggressiveness and impairment of both tumor invasion and infiltration of the skin. [Val^4^,Gln^5^]dDAVP reduced in vitro proliferation and migration in aggressive V2-receptor expressing human lung (NCI-H82) and prostate (PC-3) cancer cell lines with neuroendocrine characteristics [149]. In contrast to dDAVP, treatment with [Val^4^,Gln^5^]dDAVP abolished the formation of experimental metastases in the lungs of mice [148]. [Val^4^,Gln^5^]dDAVP impaired the spread and growth of colorectal cancer cells in the liver and reduced experimental lung colonization [150]. Importantly, studies employing animal models revealed that [Val^4^,Gln^5^]dDAVP is safe and, similarly to dDAVP, is not toxic [148]. The presence of Val4 in place of Gln4 offers higher hydrophobicity, whereas Gln introduced in position 5 is considered as less susceptible to deamidation than Asn. The relevance of chirality at position 4 was confirmed. Moreover, it was shown that tetrapeptides corresponding to the conformational loop of dDAVP (fragment 2–5 flanked with Cys residues) display similar antiproliferative effects on MCF-7 cell cultures as longer, parental peptides [145].

Wiśniewski et al. [151] described the quest for novel, potent and short-acting V2 receptor agonists. Interestingly, the reduction in antidiuretic duration may be of particular interest in older people with impaired function of kidneys, which are responsible for the elimination of dDAVP via passive glomerular filtration into urine. The prolonged antidiuresis may result in adverse effect known as hyponatremia (serum sodium concentration lower than 135 mmol/L). A series of *C*-terminally truncated analogues of dDAVP deprived of Gly9 amide and modified at positions 2, 3, 4 or 7 was analyzed. In some of them, the disulfide bridge was replaced with carba-thioether linkage. In order to improve systemic clearance and increase receptor selectivity, more lyophilic peptides were designed. Some novel agonists retained potent in vitro human V2 receptor activity and presented substantially improved selectivity versus the related human V1a, V1b and oxytocin receptors. The most promising results were obtained for two peptides having (*β*-(4-chlorophenyl) alanine ([Tyr(4-Cl)^2^]dDAVP, **63**, Figure 9) instead of Tyr2, *β*-(2-thienyl) alanine ([Thi^3^,Val^4^]dDAVP, **64**, Figure 9) in place of Phe3 and Val in position 4. Both have carba-thioether modification of the disulfide bridge. Agmatine was connected to one of the peptides’ *C*-terminal end. Both novel analogues produced dose-related decreases in urine output comparable to that of dDAVP. They also presented shorter duration of antidiuretic action due to their higher systemic clearance and shorter half-lives. It was concluded that a higher number of carbon atoms in the structures (thus higher lipophilicity) resulted in increased systemic clearance. It is worth noting that some compounds were cleared exclusively via non-renal mechanisms, presumably proteolysis and transport into organs [151].

dDAVP may be administered at various doses by different routes, including intravenous and subcutaneous injections (the most common single dose for a human is 2 μg), an intranasal spray (10–20 μg) and drop, orally available solid tablets (mostly between 200 and 400 μg) or sublingual melt formulation (60–240 μg, oral lyophilizate/orally disintegrating tablet) [152]. The last route is becoming more popular because orally administered lyophilizate melted under the tongue is fast dissolving, has a higher rate of bioavailability than a tablet and needs shorter time periods to reach maximal biological effect [153,154,155,156]. Lottmann et al. showed that treatment with sublingual dDAVP, which excludes the need to swallow tablets, is related to higher compliance with children aged 5–11 years [157]. Increased patient conformity (aged 5–15 years) was also documented by Juul et al. [155]. The bioavailability of dDAVP oral tablets is very low, between 0.08 and 0.16%. The bioavailability of oral administration is 5% of intranasal and 0.16% of intravenous [158]. Despite the low oral bioavailability, dDAVP exerts antidiuretic effects due to its high affinity to V2. Moreover, the concomitant food intake might have a substantial effect on dDAVP bioavailability [159]. In turn, nasal administration is burdened with high fluctuations of dosage, which can cause unexpected and dangerous side effects [160]. Despite poor bioavailability, oral delivery routes are still considered as highly demanded. Very recently, Kottke et al. [161] demonstrated the production of minitablets (smaller than 3 mm) containing the precise dosage of dDAVP, which rapidly disintegrates and provides immediate drug release. Zupančič et al. [162] showed that self-emulsifying drug delivery systems containing dDAVP are stable in vitro to glutathione and α-chymotrypsin degradation and are non-toxic. Thus, it might be considered as a novel, potential delivery strategy for oral dDAVP administration. Currently, the medical application of dDAVP includes the treatment of central diabetes insipidus, primary nocturnal enuresis and nocturia [163]. Recently, it was shown that low dose desmopressin Noqdirna^®^, as lyophilizate, is highly effective in treatments of nocturia due to idiopathic nocturnal polyuria in adults [164], same as MINIRIN^®^ MELT 1995 [165]. At much higher doses, dDAVP is used to treat coagulation disorders, such as hemophilia A and von Willebrand’s disease (VWD). Moreover, this peptide is applied to prevent excessive bleeding during surgical procedures [166]. Noteworthy, dDAVP application is considered to be safe and is correlated with few adverse effects, such as headaches, diarrhea and potentially serious conditions of hyponatremia. The last condition is feasible in adult patients with renal failures. Thus, they are exposed to prolonged antidiuretic action of dDAVP, which is mostly excreted from the body in urine [158].

As mentioned above, dDAVP is a common hemostatic agent that facilitates clotting cascades in patients with VWD and hemophilia A. In the latter disease, which is manifested with an insufficient level of clotting factor VIII in human blood, the administration of dDAVP is beneficial only in patients with detectable levels of this endogenous factor [167]. In cases of inherited bleeding disorder VWD, which is classified into several types, dDAVP is theoretically useful in the treatment of type 1 VWD (patients with functionally normal von Willebrand’s factor—vWF). In practice, however, patients react differently to dDAVP administration, and such a treatment is routinely preceded with responsiveness challenge [168,169]. dDAVP is not recommended in type 2A (patients with qualitatively abnormal factors) and even contraindicated in 2B type due to the risk of transient thrombocytopenia after its administration. In patients with type 3, there is no response due to undetectable levels of vWF [170].

dDAVP is the significantly potent and selective agonist of G protein–coupled V2 membrane receptor in rats and in humans. In the latter case, it is also a strong agonist of the V1b receptor with an even lower *K*i value than compared to the V2 receptor [121]. Nevertheless, V2 is mostly considered as the main pharmacological target for dDAVP. The functional extrarenal expression of V2 was also demonstrated in lung endothelial cells [171,172], as well as in many types of cancer cells, including lung, colorectal and breast cancer [173,174,175,176]. dDAVP, as a strong agonist of V2, has been repurposed as an adjuvant agent in the treatment of various tumors. It was reported to reduce tumor-induced angiogenesis in the breast cancer model [177]. The intravenous administration of dDAVP into Balb/c mice bearing aggressive F3II mammary carcinoma, resulted in an increased formation of angiostatin by tumor cells. The enhanced formation of angiostatin, which is a potent natural inhibitor of angiogenesis produced by cancer-mediated proteolysis of plasminogen, is likely associated with dDAVP-induced secretions of plasminogen activators such as uPA. As described by Gately et al. [178] such activators participate in the generation of angiostatin.

After the promising results of studies with mouse models [179,180] and two different veterinary trials in dogs with locally advanced mammary tumors [181,182], which demonstrated a significant reduction in metastatic progression and survival benefits, a phase II dose-escalation trial (NCT01606072) was conducted [183]. In that research, dDAVP (lyophilized formulation in saline) was administered intravenously to 20 patients with breast cancer before and after surgical resection. As a result, reduced intraoperative bleeding and increased plasma level of vWF was reported. Noteworthy, the growth of vWF secretion is beneficial for both hemostatic [184] and antimetastatic activity [185]. Additionally, as was detected by quantitative real-time reverse transcription-PCR assay for the expression of cytokeratin-19 mRNA in whole blood, a short postoperative drop in circulating tumor cells counts was observed. After one month, the level of cytokeratin-19 transcript returned to the baseline detected before surgery. The next phase I/II trial (NCT01623206) demonstrated that dDAVP may be considered as a promising hemostatic agent in rectal cancer patients with bleeding [186]. After a short, two-day treatment with dDAVP most patients showed at least a partial hemostatic response while about 60% had complete absence of bleeding symptoms at fourth day after the treatment started. Such an effect was observed at the last follow-up on day 14. Perfusion of rectal tumor was reduced in two-thirds of patients after dDAVP administration. The most prominent treatment-related severe adverse event was hyponatremia, associated in some cases with increased blood pressure.

Very recently, Sobol et al. [187] showed for the first time that dDAVP exerts antitumor in vivo activity on MG-63 human osteosarcoma xenografts in immunocompromised mice. Upon sustained intravenous administration, a significant reduction in tumor volume over time was reported. As compared to the control group, 34% tumor growth rate inhibition and a 25% reduction in resected tumor weight were observed. As examined by body weight and blood biochemical and hematological analyses, the antitumor action was accompanied by good tolerability and safety. Interestingly, the inhibition of phosphodiesterases, which are responsible for cAMP degradation, by rolipram enhanced the antiproliferative effects of dDAVP in osteosarcoma cells [166].

A combination treatment of dDAVP with the cytotoxic agent, docetaxel, resulted in a significant reduction in the proliferation of castrate-resistant prostate cancer (CRPC) cell line DU145 [188]. Moreover, dDAVP enhanced the anti-migratory effect of the drug and increased the inhibition of tumor growth in a xenograft model of prostate cancer (the mean tumor volume was about 41% lower as compared to treatment with the docetaxel alone). The effectiveness of that combined therapy was also confirmed using an in vivo orthotopic mouse model of CRPC [189].

##### Selepressin

Selepressin (**55**, Figure 8) [125] is another synthetic peptide analogue of AVP in which the following substitutions were applied Tyr2 → Phe2, Phe3 → Ile3, Gln4 → hGln4 and Arg8 → Orn(*i*Pr)8 (Table 4). Selepressin may be used in the treatment of patients with septic shock during sepsis [125,127]. Sepsis is characterized by vasodilation and increased capillary permeability, which results in hypotension and loss of intravascular fluid and, subsequently, death [125,127]. The main causes of its mortality are low endothelial permeability and vascular hyperpermeability, which lead to organ dysfunction [126]. Thrombin, vascular endothelial growth factor (VEGF), angiopoietin 2 and LPS-induced pulmonary microvascular endothelial barrier disruptor cause endothelial dysfunction. Selepressin counteracts the effects of these disruptors and improves endothelial barrier function [126]. It also upregulates the expression of *p53* (tumor suppressor gene), which is involved in the enhancement of the endothelial barrier. Selepressin is a potent vasopressor and it is selective for the V1a receptor [190,191]. The latter was demonstrated during phase I clinical trials in humans, when selepressin showed V1a-agonistic vasopressor properties and no signs of V2 activity [192,193]. In the phase IIa randomized, placebo-controlled trial in patients with septic shock, selepressin maintained adequate arterial pressure in the absence of norepinephrine and increased the proportion of patients that no longer require mechanical ventilation [127,193]. In phase IIb-III, selepressin was tested in vasopressor-dependent septic shock patients for its ability to reduce the duration of vasopressor and mechanical ventilator treatment. The administration of selepressin did not cause improvements compared with placebos, and the testing phase was terminated [192].

The present study indicates that the application of selepressin may be a potential alternative to AVP and serve as a supplementary vasopressor, especially to increase arterial pressure, prevent microvascular leak and reduce pulmonary edema [127]. However, further research is needed to confirm the role of selepressin.

##### Felypressin

Felypressin, also called octapressin, (**56**, Figure 8, Table 4) is known to have potent vasoconstricting properties and low toxicity. It works on smooth muscles [128], is able to constrict coronary vessels [194] and is an antidiuretic agent [129], although the last two activities are weaker than in the case of the parent peptide, AVP.

Because of its vasoconstricting properties, felypressin is used in anesthesia in dentistry and medicine. It promotes local vasoconstriction, thus enhancing the painkilling effect and decreasing bleeding during surgery [195,196,197]. It is used as a replacement of epinephrine and adrenaline due to fewer side effects [198,199] and higher median lethal dose (LD50) [200]. Inagawa et al. [130] studied the effect of epinephrine and felypressin on myocardial oxygen balance when applied in dental anesthesia at routine doses. It was found that felypressin is responsible for lowering myocardial tissue oxygen tension, heart rate and aortic and myocardial tissue blood flow. In the case of epinephrine, both aortic and myocardial tissue blood flows increased, while myocardial tissue oxygen tension remained unchanged.

Even though felypressin has a hemodynamic effect, the amount that is applied during dental procedures is too small to observe any disturbances [198] for hypertensive patients [201]. Prilocaine with felypressin is an anesthetic agent of choice even in case of very high-risk patients suffering multiple disorders [202]. Interestingly, when the influence of prilocaine/felypressin anesthetic on the autonomic nervous system during extraction of impacted mandibular third molar was compared to lidocaine/adrenaline, the differences in circulation dynamics were observed. It was explained by increased sympathetic nervous activity in case of the first and a decrease in parasympathetic nervous activity in case of the second local anesthetic agent [203]. Prilocaine/felypressin application is also recommended for adults over 65 years old in cases when tachycardia needs to be avoided [204]. Despite the general safety of felypressin, animal studies indicated myocardial ischemia [130] and, in some cases, reduced coronary blood flow [131] caused by this peptide.

##### Ornipressin

Ornipressin is an AVP analogue where Arg2 is replaced with Orn (**57**, Figure 8, Table 4). Similarly to other AVP analogues, it results in vasoconstriction and acts on microcirculation. The application of vasoconstrictors before laparoscopic myomectomy has already been recommended [136]. Ornipressin half-life is 1–2 h and, indeed, Assaf et al. [137] reported reduced blood loss during laparoscopic myomectomy if ornipressin was applied.

Similarly, during minimally invasive myomectomy, ornipressin was reported to significantly reduce blood loss and the need for transfusion and only oxytocin was observed to provide better results in the ranking score of treatments (*p*-score = 0.92 vs. *p*-score = 0.93, respectively) [138]. Additionally, ornipressin was the most successful in shortening hospital stay but only when combined with tranexamic acid (TXA). Interestingly, if applied alone, it was one of the least effective.

Vasoconstrictors, including ornipressin (similarly to terlipressin), proved to be useful also in liver cirrhosis if hepatorenal syndrome (HRS) was diagnosed [205]. HRS is caused primarily by systemic circulatory dysfunction, but cirrhotic cardiomyopathy and systemic inflammation also contribute to this renal failure [206].

##### Terlipressin

Terlipressin (glypressin, *N*-tryglycyl-8-lysine-vasopressin, **58**, Figure 8, Table 4) is a pro-drug for the endogenous/natural porcine hormone LVP [87,132]. It is converted in the human body to its active metabolite, LVP, when three *N*-terminal glycines are cleaved by endothelial peptidases. Terlipressin is reported as a long-acting AVP analogue. Its plasma half-life is 240–360 min [64] and the compound reaches the highest concentration in plasma 60–120 min after administration. Drug metabolism is mediated by endo- and exopeptidases. Only about 1% of the drug is excreted unchanged by the kidneys [133,134]. The main mechanism of its action is to stimulate V1 receptors located predominantly in vascular smooth muscle cells within the splanchnic circulation, resulting in splanchnic vasoconstriction. The terlipressin-induced splanchnic vasoconstriction induces increased renal blood flow and has beneficial effects on HRS. It also reduces portal pressure and plays a role in reducing the risk of portal hypertensive bleeding [135,207].

Terlipressin also has a trace ability to stimulate V2 [208]. For this reason, drug-induced hyponatremia should be taken into account in patients receiving terlipressin [209,210,211]. There is also experimental evidence that the stimulation of V2 vasopressin receptors causes dilation of the arcuate vessels of the kidneys with an increase in blood flow in the parenchymal layer of the kidney [212]. The myoconstrictive effect of terlipressin affects not only the vascular muscle but also the smooth muscles outside the bloodstream. It results, inter alia, in an increase in the peristalsis of the esophagus, stomach and intestines, as well as in the intensification of uterine contractile activity [207].

Apart from the direct influence on V1 receptors, other indirect effects of terlipressin’s vasoconstricting action are also postulated. One of them is the inhibition of the production of nitric oxide by the endothelium [213]. Another one is the blocking of ATP-dependent potassium channels found in vascular smooth muscle during in vitro studies [214]. The visceral vasoconstriction and reduced blood flow to the portal vein reduce portal pressure, including a decrease in pressure in esophageal varices, while increasing total hepatic blood flow [215,216]. Terlipressin infusion reduces blood pressure in oesophageal varices by 35%, and the effect is greater when the portal pressure is higher [217,218,219]. Due to these properties, terlipressin is a drug recommended in the treatment of varicose bleeding in patients with liver cirrhosis [220,221,222]. Terlipressin is much better tolerated in liver cirrhosis than AVP; however, it may cause side effects, especially in the group of patients with cardiac burden. When using terlipressin, stenocardial complaints may be intensified. On the other hand, complications such as hypertensive crisis, dangerous cardiac arrhythmias and acute limb or intestinal ischemia are less frequent [221]. Patients treated with terlipressin may complain of abdominal pain, diarrhea, foot pain or bradycardia [222].

The unique pharmacological effects of terlipressin on circulation in cirrhosis include the reduction in portal pressure and increase in renal blood flow. These effects have been exploited in studies exploring its role in the management of HRS and variceal bleeding [207,223]. In recent years, as the physiological effects of terlipressin became better understood, roles of the drug in the setting of refractory ascites and cirrhotic hyponatremia have been proposed [224,225,226]. Despite the fact that terlipressin has been used for over 20 years, research on this drug is still continued. There are currently more than 50 ongoing clinical studies analyzing the further expansion of the role of this drug. For clinicians involved in the management of patients with advanced liver disease, terlipressin plays a central role in the management of complications and its participation is likely to expand in the coming years [135].

To overcome the rapid clearance of the polypeptide from blood circulation and its in vivo instability, Wang et al. [227] utilized chemical modifications of terlipressin with polyethylene glycol (PEG). The reaction was conducted in different pH value buffers at different molar ratios and PEGylation degree and position were determined. It turned out, that the highest amount of mono-PEG-terlipressin was obtained in the reaction between terlipressin and succinimidyl propionate-monmethoxy polyethylene glycol (m-PEG-SPA) performed in the lower content of PEG and in low pH value. Synthesis of di-PEG-terlipressin was the most effective at higher pH values and higher contents of PEG [227]. Furthermore, mono-PEG-terlipressin showed proteolytic cleavage during tryptic digestion, while di-PEG-terlipressin was resistant to it.

### 2.2. AVP and Its Analogues in Treatment of SARS-CoV-2

In 2019, a new severe acute respiratory syndrome coronavirus 2 (SARS-CoV-2) appeared and caused a new disease: COVID-19 [228]. The pathophysiological role of SARS-CoV-2 infection is mediated by abnormal immune response, endothelial dysfunction, direct viral toxicity and thrombo-inflammation, which lead to pulmonary manifestation [229]. Since the beginning of the pandemic, more attention has been focused on people with increased risk of morbidity and mortality caused by different comorbidities. Long-term exposure to SARS-CoV-2 infection leads to complex health problems in endocrine and cardiovascular systems [230]. Furthermore, it was shown that body functions regulated by AVP (regulation of the blood osmotic system, blood pressure, plasma volume and body water content) are disrupted during COVID-19 and related with poor clinical outcomes [229]. Therefore, the investigation of the relationship between AVP or its analogues and SARS-CoV-2 infection became high priority.

**Figure 10:**
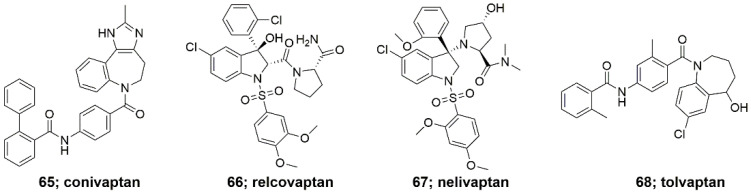
AVP receptor antagonists.

AVP was proved to be produced in COVID-19 patients in response to fever, dehydration, pain, physiological stress and high concentrations of pro-inflammatory cytokines, in order to oppose high blood viscosity [229]. High levels of AVP results in hyponatremia and inflammatory disorder. Its low concentrations, it leads to immunomodulatory effects, mainly in the lungs, through V2 receptor and further to the development of complications in COVID-19. AVP is present in the immune cells and can be released in response to inflammation and stress. AVP receptors are expressed on the immune cells involved in the release of pro-inflammatory cytokines and antibody production. They can be inhibited by vasopressin receptor antagonists (VRAs). These include the following: non-selective conivaptan (**65**, Figure 10) (blocks V1a and V2 receptors), V1a receptor antagonist relcovaptan (**66**, Figure 10), V1b receptor antagonist nelivaptan (**67**, Figure 10) and V2 receptor antagonist tolvaptan (**68**, Figure 10). In addition to the inhibition of AVP receptors, an in silico study showed that conivaptan inhibits SASRS-CoV-2 3C-like protease and viral RNA-dependent polymerase. In addition, it revealed that tolvaptan has anti-inflammatory and anti-fibrotic effects through the inhibition of monocyte chemotractic protein-1 and transforming growth factor *β*1, which are involved in the inflammatory process during SARS-CoV-2 infection. Therefore, AVP antagonists are considered as potential therapeutic agents for SARS-CoV-2 infection treatment.

Various in silico studies confirmed the potential of AVP analogues in COVID-19 treatment. Maffucci and Contini [231], in their recent work, used virtual screening (VS) to facilitate drug repurposing against SARS-CoV-2, targeting viral main proteinase and spike protein with 3000 existing drugs. The VS campaign on Spike protein Receptor Binding Domain (RBD) indicated that pressure regulators, i.e., terlipressin and lypressin, identified herein as potential binders of the S-protein may also be evaluated against SARS-CoV-2. Lypressin’s and ornipressin’s potential in the treatment of this intricate infection was confirmed. They were reported to have strong binding activity to both single chain core and the complex form (holoenzyme) of the SARS-CoV-2 RNA dependent RNA polymerase (RdRp) [232]. A docking study revealed a docking score of −11.717 kcal/mol for ornipressin and −11.923 kcal/mol for lypressin. Due to potent interaction with both forms of RdRp, the abovementioned AVP analogues are suggested to be tested as potential treatments of SARS-CoV-2 infection.

In addition, Sheikh et al. [230] reported a case of a patient who had a high serum sodium level and low urine osmolality, which were symptoms of diabetes insipidus developed after long-term exposure to the virus. Upon the administration of 2 µg of desmopressin, the patient improved clinically and symptomatically.

## 3. Conclusions

In this review, we presented the most relevant information regarding arginine vasopressin (AVP) and its analogues. AVP acts on three different G protein-coupled receptors (V1a, V1b and V2) and, depending on the interaction, demonstrates different functions. They include vasoconstriction, glycogenolysis, modulation of ACTH synthesis, stimulation of water reabsorption, insulin secretion, regulation of blood pressure, etc. Its expression in the brain acts on social interaction, depression and aggression. The first isolation, sequencing and chemical synthesis of AVP, conducted in the 1950s by du Vigneaud and his team, is deemed to be a landmark in organic chemistry. The subsequent identification of its receptors in the 1980s and 1990s gave rise to further exploration and understanding of biological properties displayed by this peptide hormone, as well as its natural (lypressin and phenypressin) and synthetic analogues (desmopressin, selepressin, felypressin, ornipressin and terlipressin) and many other synthetic non-peptide and peptide analogues.

Despite its extensive application in medicine, the use of AVP still has serious limitations, such as short biological half-life and lack of specificity for receptors resulting in side effects (e.g., hyponatremia, decrease of cardiac output and platelet count). In addition to AVP, its several efficient and relatively safe analogues were approved as medications. Felypressin is successfully used as anesthesia in medicine and dental care. Terlipressin is recommended in the treatment of varicose bleeding in patients with liver cirrhosis. dDAVP is utilized in the treatment of polyuric conditions including primary nocturnal enuresis, nocturia and diabetes insipidus as well as coagulation disorders, such as hemophilia A and von Willebrand’s disease. Compared to AVP, non-peptide analogue belovaptan showed amelioration of socialization and communication. Peptides lypressine (pigs) and phenypressin (marsupials) are characterized by easier applications in the treatment of some diseases, while having the same or better effect than AVP. Moreover, the interaction of analogues of AVP and LVP with receptors helped in the determination of key amino acids residues for their function and selectivity. It was shown that the activity and selectivity of AVP analogue responses to receptors depend on the steric size of group at the key positions (two, three, seven and eight) in peptides. The steric size of the side chain in position three of LVP plays significant role in the manifestation of vasopressin-like activities, but is not crucial for selectivity towards receptors. As in the case of many other known drugs, AVP and its analogues also were subjected to a repurposing strategy aimed at finding their novel applications. In this manner, desmopressin analogue [Val4,Gln5]dDAVP was found to possess not only antidiuretic but also anti-cancer activity. Lypressin, ornipressin and desmopressin showed satisfactory results in the treatment of SARS-CoV-2.

For several decades, exogenous AVP has been administered in the treatment of various diseases, including variceal bleeding, diabetes insipidus and vasodilatory shock. However, its non-specific binding to V1 and V2 receptors may promote potentially dangerous side effects. Even though many attractive AVP substitutes were developed and successfully applied in medicine, a quest for novel, highly specific AVP analogues remains an area of great interest. The search for AVP counterparts presenting either agonistic or antagonistic properties, characterized with proper ratio of structural complexity, activity and low toxicity, is still an open field.

## Figures and Tables

**Table 4:** Synthetic analogues of AVP. Amino acid residues that are not present in AVP are marked in bold.

Analogue	Sequence	Functions	Refs.
Desmopressin, dDAVP	dCys(&)-Tyr-Phe-Gln-Asn-Cys(&)-Pro-d-**Arg**-Gly-NH_2_	antidiuretic effect, increases plasma osmolality	[64,120,121,122]
Selepressin	Cys(&)-**Phe**-**Ile**-**hGln**-Asn-Cys(&)-Pro-**Orn(*i*Pr)**-Gly-NH_2_	applied in septic shock	[125,126,127]
Felypressin	Cys(&)-**Phe**-Phe-Gln-Asn-Cys(&)-Pro-**Lys**-Gly-NH_2_	vasoconstricting agent, used as an additive in anesthesia during dental procedures	[128,129,130,131]
Terlipressin	**Gly**-**Gly**-**Gly**-Cys(&)-Tyr-Phe-Gln-Asn-Cys(&)-Pro-**Lys**-Gly-NH_2_	treats bleeding caused by esophageal varices	[132,133,134,135]
Ornipressin	Cys(&)-Tyr-Phe-Gln-Asn-Cys(&)-Pro-**Orn**-Gly-NH_2_	vasoconstricting agent during myomectomy; in cirrhosis, as hepatorenal treatment	[136,137,138]
